# Lipid Emulsion Inhibits the Late Apoptosis/Cardiotoxicity Induced by Doxorubicin in Rat Cardiomyoblasts

**DOI:** 10.3390/cells7100144

**Published:** 2018-09-20

**Authors:** Raghavendra Baregundi Subbarao, Seong-Ho Ok, Soo Hee Lee, Dawon Kang, Eun-Jin Kim, Ji-Yoon Kim, Ju-Tae Sohn

**Affiliations:** 1 Department of Anesthesiology and Pain Medicine, Gyeongsang National University School of Medicine and Gyeongsang National University Hospital, Jinju-si 52727, Korea; raghu_dvs@yahoo.com (R.B.S.); lishiuji@naver.com (S.H.L.); 2 Institute of Health Sciences, Gyeongsang National University, Jinju 52727, Korea; mdoksh@naver.com; 3 Department of Anesthesiology and Pain Medicine, Gyeongsang National University Changwon Hospital, Changwon 51427, Korea; 4 Department of Physiology, Gyeongsang National University School of Medicine, Jinju-si 52727, Korea; dawon@gnu.ac.kr (D.K.); eunjin1981@hanmail.net (E.-J.K.); 5 Department of Anesthesiology and Pain Medicine, Gyeongsang National University Hospital, Jinju-si 52727, Korea; avadore33@naver.com

**Keywords:** lipid emulsion, doxorubicin, apoptosis, cardiotoxicity, cardiomyoblasts

## Abstract

This study aimed to examine the effect of lipid emulsion on the cardiotoxicity induced by doxorubicin in H9c2 rat cardiomyoblasts and elucidates the associated cellular mechanism. The effects of lipid emulsion on cell viability, Bax, cleaved caspase-8, cleaved capase-3, Bcl-XL, apoptosis, reactive oxygen species (ROS), malondialdehyde, superoxide dismutase (SOD), catalase and mitochondrial membrane potential induced by doxorubicin were examined. Treatment with doxorubicin decreased cell viability, whereas pretreatment with lipid emulsion reduced the effect of doxorubicin by increasing cell viability. Lipid emulsion also suppressed the increased expression of cleaved caspase-3, cleaved caspase-8, and Bax induced by doxorubicin. Moreover, pretreatment with lipid emulsion decreased the increased Bax/Bcl-XL ratio induced by doxorubicin. Doxorubicin-induced late apoptosis was reduced by treatment with lipid emulsion. In addition, pretreatment with lipid emulsion prior to doxorubicin enhanced glycogen synthase kinase-3β phosphorylation. The increased malondialdehyde and ROS levels by doxorubicin were reduced by lipid emulsion pretreatment. Furthermore, lipid emulsion attenuated the reduced SOD and catalase activity and the decreased mitochondrial membrane potential induced by doxorubicin. Taken together, these results suggest that lipid emulsion attenuates doxorubicin-induced late apoptosis, which appears to be associated with the inhibition of oxidative stress induced by doxorubicin.

## 1. Introduction

Doxorubicin, an anticancer drug extracted from *Streptomyces peucetius var Caesius*, has been shown to be highly effective against various human malignancies [1]. However, its successful use in clinical cancer treatment has been limited due to its association with cardiotoxicity. Doxorubicin is known to induce cardiotoxicity in a dose-dependent manner, while it exerts different effects across individuals based on gender, age and time of cardiotoxicity onset [1]. Complex mechanisms underlie doxorubicin-induced cardiotoxicity, one of which is associated with reactive oxygen species (ROS)-induced apoptosis in mitochondria, which leads to congestive heart failure [2,3,4]. Many reports have shown that doxorubicin accumulates in cardiomyocyte mitochondria at a concentration one hundred times higher than that in the extracellular space [5,6]. Lipid emulsion, which was originally developed for parenteral nutrition, is now also used clinically as drug delivery vehicles (for example, propofol and etomidate) that do not induce severe side effects [7]. In addition, lipid emulsion is effective at treating systemic toxicity induced by local anesthetics and other drugs with high lipid solubility [8]. Lipid nanoemulsion with doxorubicin and oleic acid reduces the doxorubicin concentration in the heart and increases the blood concentration of doxorubicin [9]. Furthermore, α-linolenic acid, a long-chain fatty acid present in Intralipid^®^, inhibits the cardiotoxicity induced by doxorubicin [10]. However, as intravenously administered fats do not pass through the gastrointestinal tract, these fats do not undergo pancreatic lipase-induced hydrolysis, bile-induced emulsification or transformation into chylomicron [11]. Thus, in contrast to fatty acids, lipid emulsions for intravenous administration are prepared to be similar to the structure of chylomicron delivered into a hydrophilic environment, and lipid emulsions contain triglycerides (one glycerol plus three fatty acids), phospholipids (an emulsifier), and glycerin (an osmotic agent) [11]. The mechanisms underlying lipid emulsion treatment include the scavenging effect, the inotropic effect, fatty acid supply, reversal of mitochondrial dysfunction, glycogen synthase kinase (GSK)-3β phosphorylation, inhibition of nitric oxide release, and attenuation of a cardiac sodium channel blockade [8]. Moreover, lipid emulsion attenuates lung injury induced by acute malathion toxicity via reducing oxidative stress [12]. Based on these previous reports, we tested the hypothesis that lipid emulsion attenuates doxorubicin-induced apoptosis by inhibiting oxidative stress [2,3,4,8,10,12]. The goal of this in vitro study was to examine the effect of lipid emulsion (Intralipid^®^ 20%) on the apoptosis induced by doxorubicin in H9c2 rat cardiomyoblasts and to elucidate the associated cellular mechanism.

## 2. Materials and Methods

All experimental methods and protocols were performed in accordance with the Regulation for the Care and Use of Laboratory Animals stipulated by Gyeongsang National University.

### 2.1. Cell Culture

H9c2 rat cardiomyoblasts obtained from the American Type Culture Collection (Manassas, VA, USA) were cultured in Dulbecco’s modified Eagle’s medium (DMEM, HyClone, GE Healthcare, Salt Lake City, UT, USA) supplemented with 10% fetal bovine serum, 100 U/mL penicillin, and 100 mg/mL streptomycin as described previously [13,14]. Cells were cultured in 100-mm dishes at 37 °C in 5% CO_2_ and sub-cultured in 1:4 ratios upon reaching 90% confluence. All subsequent experiments were conducted using cells of at least passage 3.

### 2.2. Cell Viability Assay

Cell viability was estimated colorimetrically using the 3-(4,5-dimethylthiazole-2-yl)-2,5-diphenyl tetrazolium bromide (MTT) assay as previously reported [13,14]. Cells were seeded on a 24-well plate at a density of 10^5^ cells/well and were treated with doxorubicin (10^−6^, 3 × 10^−6^ and 10^−5^ M) alone for 24 h, treated with lipid emulsion (0.125, 0.25, 0.75 and 2%) alone for 25 h, or pretreated with lipid emulsion (0.125, 0.25, 0.75 and 2%) for 1 h followed by doxorubicin (10^−5^ M) for 24 h. After incubation, the cells were washed twice with phosphate buffered saline (PBS, pH 7.4), treated with MTT in PBS (0.5 mg/well), and then incubated for 4 h at 37 °C in the dark. After incubation, the MTT solution was removed, and the formazan crystals formed in each well were dissolved in 200 μL of dimethyl sulfoxide for 20 min at 37 °C under gentle shaking. The absorbance was measured spectrophotometrically using VersaMax^®^ (Molecular Devices, Sunnyvale, CA, USA) at 570 nm. Untreated cells were used as controls. Intralipid^®^ (20%), used in this study, is composed of 20% soya bean oil, 1.2% egg yolk phospholipid, 2.25% glycerin and water for injection [15]. The fatty acid composition of Intralipid^®^ includes linoleic, oleic, palmitic, linolenic and stearic acid [15].

### 2.3. Apoptosis Assay

#### 2.3.1. Annexin V-FITC-PI Staining

To detect the apoptosis induced by doxorubicin in H9c2 cells, a FITC Annexin V Apoptosis Detection Kit (Invitrogen-Life Technologies, Carlsbad, CA, USA) was used, and a flow cytometric analysis was performed. Briefly, cells were cultured in 6-well plates at 3 × 10^5^ cells/well and were then treated with doxorubicin (10^−5^ M) alone for 6 h, treated with lipid emulsion (0.25%) alone for 7 h, or pretreated with lipid emulsion (0.25%) for 1 h followed by doxorubicin (10^−5^ M) for 6 h. Following the treatment, the cells were washed three times with cold PBS (pH 7.4), resuspended in 1× binding buffer and stained with Annexin V-FITC and propidium iodide (PI) for 10 min at room temperature in the dark. Finally, the cells were resuspended in 400 μL of 1× binding buffer and immediately analyzed by flow cytometry. The cell viability and apoptosis rates were measured on a FC-500 flow cytometer (Beckman Coulter, Pasadena, CA, USA) using 488-nm laser excitation and fluorescence emission at 530 nm (FL1) and >575 nm (FL3). A total of 20,000 cells per treatment condition were acquired from three independent experiments and analyzed using Beckman Coulter CPX software (Beckman Coulter, CXP 2.2, Mervue Business Park, Mervue, Galway, Ireland). For forward and side scatter measurements, linear amplification was applied, and logarithmic amplification was used for all fluorescence measurements. Quadrant analysis was performed on the gated fluorescence dot plot to quantify the percentages of live, necrotic and apoptotic cell populations.

#### 2.3.2. TUNEL Assay for Late Apoptosis Detection

To detect whether doxorubicin induces late apoptosis in H9c2 cells, a terminal deoxynucleotidyl transferase dUTP nick end-labeling (TUNEL) assay was conducted as previously reported [16,17]. Briefly, 10^5^ cells/well were cultured on coverslips in a 24-well plate and then treated with doxorubicin (10^−5^ M) alone for 6 h, treated with lipid emulsion (0.25%) alone for 7 h, or pretreated with lipid emulsion (0.25%) for 1 h followed by doxorubicin (10^−5^ M) for 6 h. After treatment, a TUNEL assay was performed using a TUNEL kit (Roche, Indianapolis, IN, USA) according to the manufacturer’s instructions, and cells were counterstained with 4′,6-diamidino-2-phenylindole (Sigma Chemical Company, St. Louis, MO, USA). TUNEL-positive cells emitting fluorescence were visualized under a Fluoview 500 fluorescence microscope (Olympus, Tokyo, Japan). Three nonoverlapping fields per coverslip were counted per treatment, and the number of TUNEL-positive cells was calculated.

### 2.4. Estimation of ROS Generation

ROS generation was measured using the fluorescent dye 2,7-dichlorodihydrofluorescein diacetate (DCFH-DA) after treatment with doxorubicin. Briefly, H9c2 cells (10^6^ cells/well) were cultured in a 24-well plate and then treated with doxorubicin (10^−5^ M) alone for 1 h, pretreated with lipid emulsion (0.25%) or α-linolenic acid (10^−6^ M) for 1 h followed by doxorubicin (10^−5^ M) for 1 h, or treated with lipid emulsion (0.25%) or α-linolenic acid (10^−6^ M) alone for 2 h. After the treatment, the cells were washed three times with PBS and stained with 5 μM DCFH-DA in PBS for 30 min at room temperature in the dark. The fluorescence intensity was measured using a FC-500 flow cytometer, and the results were analyzed using Beckman Coulter CPX software (Beckman Coulter).

### 2.5. Measurement of Antioxidants

The malondialdehyde (MDA) level, superoxide dismutase (SOD) activity, and catalase activity were evaluated using commercial detection kits for the respective assays. Briefly, H9c2 cells were grown to 70% confluence and then incubated in starvation media (DMEM without FBS) overnight. Next, the cells were treated with doxorubicin (10^−5^ M) alone for 24 h, pretreated with lipid emulsion (0.25%) for 1 h, followed by doxorubicin (10^−5^ M) for 24 h, or treated with lipid emulsion (0.25%) alone for 25 h. After treatment, the cells were washed with cold PBS, adherent cells were removed, and protein was extracted according to the manufacturer’s protocol using the respective kits. The protein concentration was estimated using the Bradford method. Lipid peroxidation (MDA) was estimated using a kit (Abcam, Cambridge, United Kingdom) according to the manufacturer’s protocol, and the optical density (OD) was measured at 532 nm using a VersaMax^®^ microplate reader (Molecular Devices). The MDA concentrations in the treated samples were calculated using the formula described in the kit. Both SOD and catalase activity was detected using kits (Abcam) according to the manufacturer’s instructions. The OD was measured at 450 nm using the VersaMax^®^ microplate reader (Molecular Devices), and the activity was calculated from the assay results.

### 2.6. Western Blot Analysis

Following treatment, proteins were extracted, and the total protein concentrations were determined using the Bradford method as previously reported [13]. The sample protein from the cell lysate was mixed with 5× sodium dodecyl sulfate sample buffer (0.1 M Tris-HCl, 20% glycerol, 4% sodium dodecyl sulfate and 0.01% bromophenol blue). Aliquots of proteins (50 µg) were separated by 10% or 12% sodium dodecyl sulfate-polyacrylamide gel electrophoresis for 150 min at 80 V, and the separated proteins were electrophoretically transferred to polyvinylidene difluoride membranes at 190 mA for 2 h. The membranes were then blocked with 5% *w*/*v* nonfat dried milk or 5% bovine serum albumin in Tris-buffered saline containing Tween-20 (TBST) for 1 h at room temperature and incubated with specific primary antibodies (anti-Bax [1:500], anti-Bcl-XL [1:500], anti-cleaved caspase-3 [1:1000], anti-cleaved caspase-8 [1:1000], anti-GSK-3β [1:1000], anti-phospho-GSK-3β [1:1000], and anti-β-actin [1:2500]), which were diluted in TBST containing 5% *w*/*v* skim milk or 5% bovine serum albumin, overnight at 4 °C. After incubation, the membranes were washed 3 times with TBST and incubated with a horseradish peroxidase-conjugated anti-rabbit or anti-mouse IgG secondary antibody diluted 1:5000 in TBST containing 5% *w*/*v* skim milk for 1 h at room temperature. The membranes were washed in TBST, and the immunoreactive signals were detected using enhanced chemiluminescence (SuperSignal^®^ West Pico Chemiluminescent Substrate, Thermo Scientific, Rockford, IL, USA) and transferred onto an X-ray film (SuperRX-N Fuji Medical X-ray Film, Tokyo, Japan). The band intensity was measured using densitometry.

### 2.7. Determination of Mitochondrial Membrane Potential

JC-1 staining was employed to assess the mitochondrial membrane potential (MMP) according to the manufacturer’s protocol (Biotium, Hayward, CA, USA). Briefly, H9c2 cells (4 × 10^3^ cells/100 μL) were cultured on glass coverslips coated with poly-l-lysine and in 96-well black plates for fluorescence microscopy and fluorescence ratio detection, respectively. The cells were treated with doxorubicin (10^−5^ M) for up to 6 h or with lipid emulsion (0.25%) for 1 h followed by doxorubicin for 6 h. In addition, cells were treated with lipid emulsion alone for 7 h. The chemically treated cells were stained with 1× MMP-sensitive JC-1 reagent at 37 °C for 15 min and washed with 1× PBS. The changes in MMP were measured using a confocal laser scanning microscope equipped with a fluorescence system (IX70 Fluoview, Olympus, Tokyo, Japan). Green JC-1 monomers and red aggregates were detected with 488 nm and 529 nm lasers, respectively. The JC-1 ratio was measured using a GloMax explorer (Promega, Madison, WI, USA), and the ratio was obtained by dividing the red fluorescence value by the green fluorescence value.

### 2.8. Chemicals and Media

All chemicals, the anti-β-actin antibody and DCFH-DA were obtained from Sigma Chemical Company. Intralipid^®^ 20% was purchased from Fresenius Kabi Korea (Seoul, Korea). The anti-Bax and anti-Bcl-XL antibodies were obtained from Santa Cruz Biotechnology (Santa Cruz, CA, USA). The anti-cleaved caspase-3, anti-cleaved caspase-8, anti-GSK-3β, and anti-phospho-GSK-3β antibodies were obtained from Cell Signaling Technology (Beverly, MA, USA). Media, serum and buffers were obtained from Gibco (Invitrogen, Burlington, ON, Canada).

### 2.9. Statistical Analysis

Data are shown as the mean ± SD. The effects of lipid emulsion on the decreased cell viability, apoptosis, expression of cleaved caspase-3 and cleaved caspase-8, Bax/Bcl-XL ratio, and GSK-3β phosphorylation induced by doxorubicin were analyzed by one-way analysis of variance (ANOVA) followed by Bonferroni’s multiple comparison test. The effects of lipid emulsion on DCFH-DA, MDA, SOD, catalase and MMP induced by doxorubicin were analyzed by one-way ANOVA followed by Bonferroni’s multiple comparison test or Tukey’s multiple comparison test. A *p* value less than 0.05 was considered statistically significant.

## 3. Results

### 3.1. Effects of Lipid Emulsion on Doxorubicin-Induced Reduced H9c2 Cell Viability

**Figure 1:**
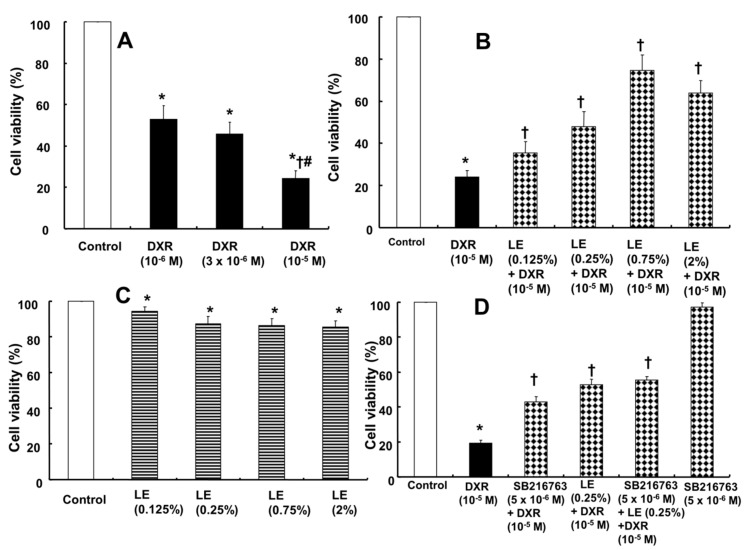
Effects of Intralipid^®^ (lipid emulsion, LE) and SB216763 alone and in combination on the doxorubicin (DXR)-induced decreased viability of H9c2 rat cardiomyoblasts as determined by the MTT assay; the results are expressed as a percentage of the untreated control group (100%). (**A**) Treatment with DXR (N = 6) at various concentrations for 24 h. (**B**) Pretreatment with 20% LE (N = 12; 0.125%, 0.25%, 0.75%, 2%) for 1 h followed by DXR (10^−5^ M) for 24 h. (**C**) Treatment with LE (N = 6) alone at 0.125%, 0.25%, 0.75%, and 2% for 25 h. (**D**) Cells (N = 6) were treated with DXR alone for 24 h; treated with SB216763 (5 × 10^−6^ M) for 1 h or LE (0.25%) for 1 h followed by DXR for 24 h; pretreated with SB216763 (5 × 10^−6^ M) for 1 h, LE (0.25%) for 1 h, and then DXR for 24 h; or treated with SB216763 (5 × 10^−6^ M) alone for 26 h. Data are presented as the mean ± SD. A and C: * *p* < 0.001 versus control. † *p* < 0.001 versus DXR (10^−6^ M). # *p* < 0.001 versus DXR (3 × 10^−6^ M). B and D: * *p* < 0.001 versus control. † *p* < 0.001 versus DXR alone.

H9c2 cell viability was reduced by doxorubicin treatment in a dose-dependent manner as assessed by the MTT assay (Figure 1A). H9c2 cells were treated with different concentrations of doxorubicin (10^−6^ M, 3 × 10^−6^ M and 10^−5^ M for 24 h), and dose-dependent decreases in cell viability of 52.84 ± 6.81%, 45.93 ± 5.59%, and 24.24 ± 3.95%, respectively, were observed compared with the cell viability of control cells (Figure 1A). Pretreatment with lipid emulsion (1 h) at different concentrations (0.125%, 0.25%, 0.75%, and 2%) before administration of the highest dose of doxorubicin (10^−5^ M) for 24 h improved the cell viability by 35.56 ± 5.23%, 48.01 ± 7.19%, 74.50 ± 7.45%, and 63.95 ± 5.77%, respectively, compared with cells treated with doxorubicin alone (24.12 ± 3.11%) (Figure 1B). However, cells treated with lipid emulsion alone (0.125%, 0.25%, 0.75%, and 2%) showed slightly decreased cell viability (94.37 ± 2.41%, 87.37 ± 4.00%, 86.34 ± 3.86%, and 85.50 ± 3.41%) compared with untreated control cells (Figure 1C). Combined pretreatment with the GSK-3β inhibitor SB216763 followed by doxorubicin increased the cell viability more than was achieved with doxorubicin alone (Figure 1D). In addition, the magnitude of the cell viability increase induced by the combined SB216763 and lipid emulsion pretreatment (0.25%) was not significantly different from that induced by lipid emulsion (0.25%) pretreatment alone (Figure 1D). These results prove that pretreatment with lipid emulsion increases the cell viability of rat cardiomyoblasts and suggest that this increased cell viability may be partially associated with the inhibition of GSK-3β.

### 3.2. Doxorubicin-Induced Late Apoptosis Was Reduced by Lipid Emulsion Pretreatment in H9c2 Rat Cardiomyoblasts

**Figure 2:**
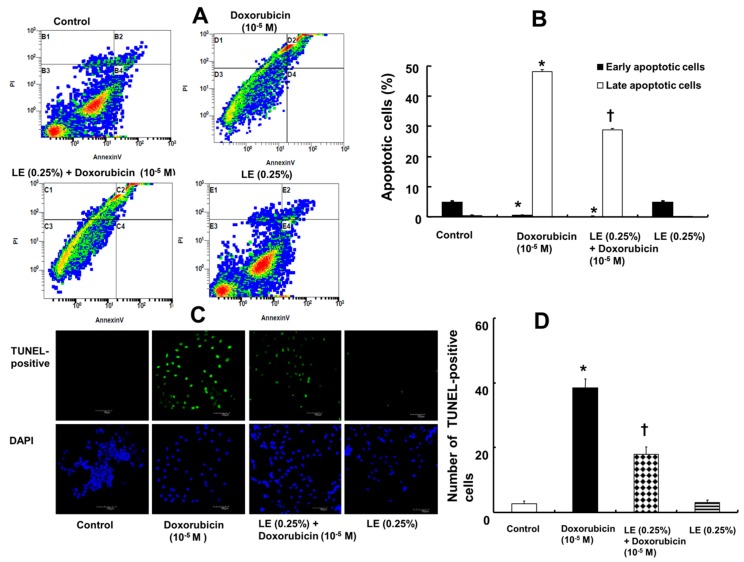
Effects of Intralipid^®^ (lipid emulsion, LE) on the doxorubicin-induced apoptosis of H9c2 rat cardiomyoblasts. The cells were pretreated with doxorubicin (10^−5^ M) alone for 6 h, LE (0.25%) for 1 h followed by doxorubicin (10^−5^ M) for 6 h, or LE (0.25%) alone for 7 h. (**A**) Annexin-V-FITC/propidium iodide staining followed by flow cytometric analysis. (**B**) Plot (N = 3) showing both early- and late-stage apoptosis after treatment. (**C**) TUNEL assay showing DNA damage after treatment as detected by immunofluorescence. Nuclei were stained with 4′,6-diamidino-2-phenylindole (DAPI) and are shown in blue. TUNEL-positive cells appear green. Scale bar: 100 μm. (**D**) Representative plot (N = 3) showing TUNEL-positive cells in different treatment groups. (**B**,**D**): Data are shown as the mean ± SD. N indicates the number of independent experiments. * *p* < 0.001 versus control. † *p* < 0.001 versus doxorubicin alone.

Doxorubicin treatment in H9c2 cells increased the percentage of late apoptotic cells as evaluated by Annexin-V-FITC/PI staining (Figure 2A) and further confirmed by the TUNEL assay (Figure 2C). Annexin-V-FITC/PI staining showed an increase in the percentage of late apoptotic cells after doxorubicin (10^−5^ M) treatment (48.11 ± 0.87%), whereas lipid emulsion pretreatment decreased the percentage of late apoptotic cells (28.89 ± 0.54%) (Figure 2B). In addition, lipid emulsion alone had no significant effect on the percentage of late apoptotic cells (0.14 ± 0.25%), which was similar to that in untreated H9c2 control cells (0.40 ± 0.28%) (Figure 2B). A TUNEL assay was performed to further confirm the apoptosis rate. The number of TUNEL-positive cells increased by doxorubicin treatment alone (38.5 ± 2.60) and was reduced by lipid emulsion pretreatment (18 ± 2.29), confirming that lipid emulsion can prevent doxorubicin-induced late apoptosis (Figure 2D).

### 3.3. Lipid Emulsion Mediated Downregulation of the Apoptotic Signaling Cascade Activated by Doxorubicin in H9c2 Rat Cardiomyoblasts

**Figure 3:**
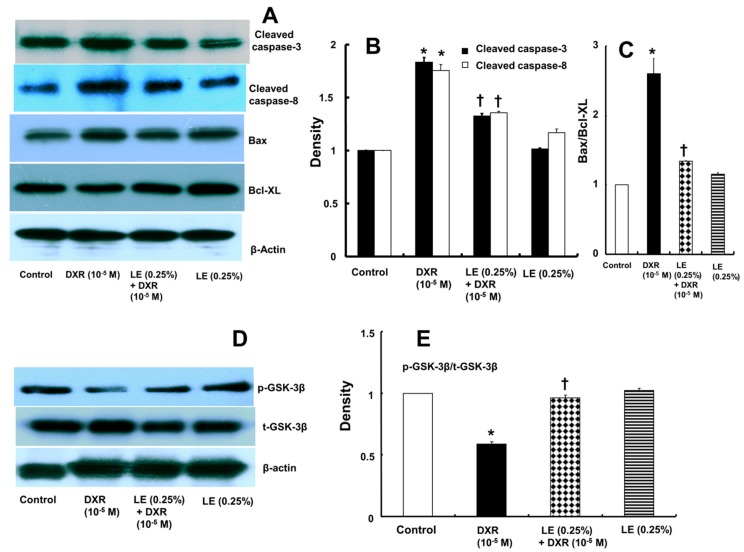
(**A**–**C**) Effects of Intralipid^®^ (lipid emulsion, LE; N = 3) on doxorubicin (DXR)-induced cleaved caspase-3, cleaved caspase-8, Bax, and Bcl-XL protein levels in H9c2 rat cardiomyoblasts. Cells were treated with DXR (10^−5^ M) for 6 h, LE (0.25%) for 1 h followed by DXR (10^−5^ M) for 6 h, or LE (0.25%) alone for 7 h. (**D**,**E**) Effect of LE (N = 3) on DXR-induced glycogen synthase kinase-3β (GSK-3β) phosphorylation. Cells were treated with DXR (10^−5^ M) for 1 h, LE (0.25%) for 1 h followed by DXR (10^−5^ M) for 1 h, or LE (0.25%) alone for 2 h. p-GSK-3β: phosphorylated GSK-3β. t-GSK-3β: total GSK-3β. β-actin was used as an internal control. (**B**,**C**,**E**) Data are shown as the mean ± SD, and all experiments were performed in triplicate. * *p*< 0.001 versus control. † *p* < 0.001 versus DXR alone.

We performed Western blot analysis to investigate changes in the expression levels of the proapoptotic protein Bax, cleaved caspase-8, cleaved caspase-3 and the anti-apoptotic protein Bcl-XL after treatment. The expression levels of Bax, cleaved caspase-8 and cleaved caspase-3 were highly increased after doxorubicin treatment alone, whereas pretreatment with lipid emulsion downregulated the expression of Bax, cleaved caspase-8 and cleaved caspase-3 (Figure 3A,B). The expression of anti-apoptotic Bcl-XL was elevated in cells pretreated with lipid emulsion followed by doxorubicin but decreased in cells pretreated with doxorubicin alone (Figure 3A). Finally, the Bax/Bcl-XL ratio was lower in cells pretreated with lipid emulsion followed by doxorubicin than in cells pretreated with doxorubicin alone (Figure 3C). These results confirm that lipid emulsion interferes with the apoptotic signaling cascade, as it downregulates proapoptotic protein expression and upregulates anti-apoptotic Bcl-XL expression by inhibiting the apoptosis induced by doxorubicin in H9c2 rat cardiomyocytes.

### 3.4. Role of Lipid Emulsion in Modulating GSK-3β Phosphorylation in H9c2 Rat Cardiomyoblasts

Doxorubicin downregulated GSK-3β phosphorylation and upregulated active GSK-3β (Figure 3D,E). However, lipid emulsion pretreatment prior to doxorubicin treatment reversed these trends by increasing GSK-3β phosphorylation and inactivating GSK-3β (Figure 3D,E).

### 3.5. Doxorubicin-Induced Oxidative Stress Was Attenuated by Lipid Emulsion Pretreatment in H9c2 Rat Cardiomyoblasts

**Figure 4:**
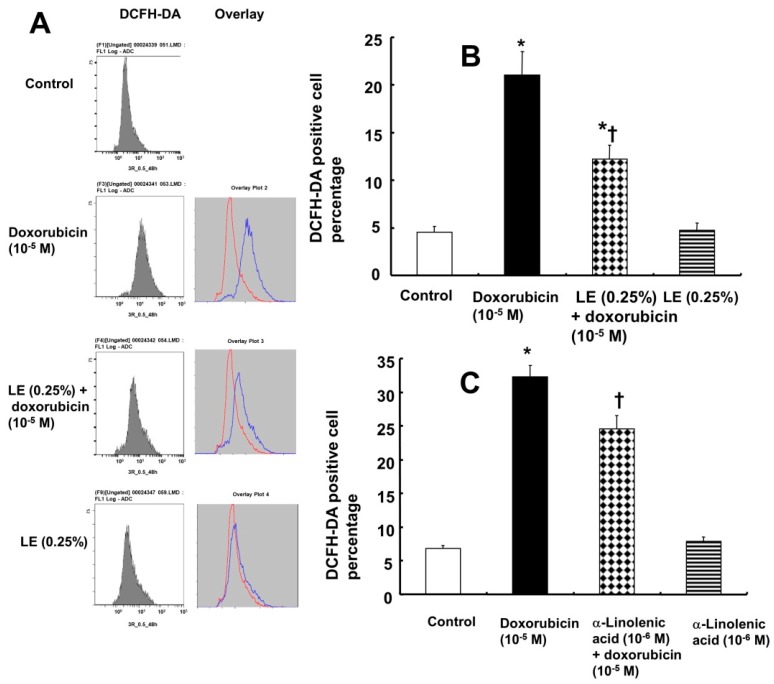
Effects of Intralipid^®^ (lipid emulsion, LE) on the doxorubicin-induced reactive oxygen species (ROS) levels in H9c2 rat cardiomyoblasts. Cellular ROS were measured by flow cytometry after staining with dichlorodihydrofluorescein diacetate (DCFH-DA) following treatment with doxorubicin (10^−5^ M) alone for 1 h, pretreatment with LE (0.25%) or α-linolenic acid (10^−6^ M) for 1 h followed by doxorubicin (10^−5^ M) for 1 h, or treatment with LE (0.25%) or α-linolenic acid (10^−6^ M) alone for 2 h. (**A**,**B**) Effect of LE on ROS levels compared with the control. (**C**) Effect of α-linolenic acid on ROS levels compared with the control. (**B**,**C**) Data are presented as the mean ± SD (N = 3). * *p*< 0.001 versus control. † *p* < 0.001 versus doxorubicin alone.

**Figure 5:**
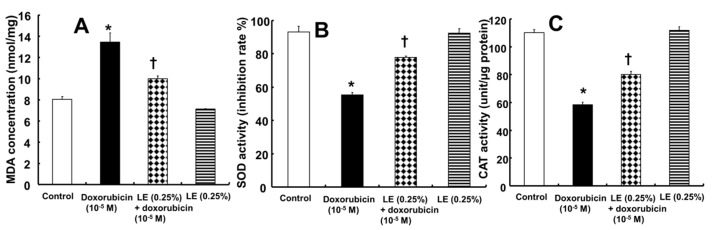
Effects of Intralipid^®^ (lipid emulsion, LE) on doxorubicin-induced oxidative stress in H9c2 rat cardiomyoblasts. H9c2 cells were treated with doxorubicin alone (10^−5^ M) for 24 h, LE (0.25%) for 1 h followed by doxorubicin (10^−5^ M) for 24 h, or LE (0.25%) alone for 25 h. The levels of malondialdehyde (MDA, **A**), a marker of lipid peroxidation, superoxide dismutase (SOD, **B**) activity and catalase (CAT, **C**) activity in different treatment groups were measured and compared with those in the control. Data are presented as the mean ± SD (N = 3). * *p*< 0.001 versus control. † *p* < 0.001 versus doxorubicin alone.

The level of ROS was evaluated after treatment with doxorubicin alone or pretreatment with lipid emulsion followed by doxorubicin treatment (Figure 4A,B). ROS accumulation in the mitochondria leads to its dysfunction, and oxidative stress is known to induce cell apoptosis [2,4]. DCFH-DA staining of ROS in H9c2 rat cardiomyoblasts indicated that doxorubicin treatment induced higher levels of ROS (21.08 ± 2.4%), and pretreatment with lipid emulsion followed by doxorubicin reduced the ROS levels (12.19 ± 1.5%) (Figure 4B). In addition, ROS production (4.75 ± 0.75%) in cells treated with lipid emulsion alone (0.25%) was not significantly different from ROS production in control cells (4.54 ± 0.63%) (Figure 4B). α-Linolenic acid (10^−6^ M) partially inhibited the increased ROS production induced by doxorubicin (Figure 4C), but treatment with α-linolenic acid alone had no significant effect on the ROS level, as compared with the control (Figure 4C). MDA, which is the product of lipid peroxidation due to oxidative stress, was increased in cells treated with doxorubicin (Figure 5A). The level of MDA (9.99 ± 0.29 nmol/mg) in cells treated with lipid emulsion followed by doxorubicin was lower than that in cells treated with doxorubicin alone (13.45 ± 0.89 nmol/mg) (Figure 5A). However, the level of MDA in cells treated with lipid emulsion alone (7.12 ± 0.067 nmol/mg) was not significantly different from that in control cells (8.03 ± 0.31 nmol/mg) (Figure 5A). These results confirm that lipid emulsion attenuates oxidative stress by downregulating the MDA level and decreasing ROS production. In addition, doxorubicin decreased SOD activity (55.3 ± 1.5%), whereas pretreatment with lipid emulsion followed by doxorubicin increased SOD activity (77.9 ± 0.66%) (Figure 5B). Lipid emulsion (92.33 ± 2.79%) alone had no significant effect on SOD activity compared with that in control cells (93.23 ± 3.37%) (Figure 5B). Furthermore, doxorubicin reduced catalase activity (58.26 ± 2.05 unit/µg protein), whereas pretreatment with lipid emulsion followed by doxorubicin increased catalase activity (80.15 ± 2.37 unit/µg protein) (Figure 5C). However, lipid emulsion alone (111.76 ± 2.92 unit/µg protein) had no significant effect on catalase activity compared with that in control cells (110.14 ± 2.48 unit/µg protein) (Figure 5C).

### 3.6. Role of Lipid Emulsion in Regulating the MMP

**Figure 6:**
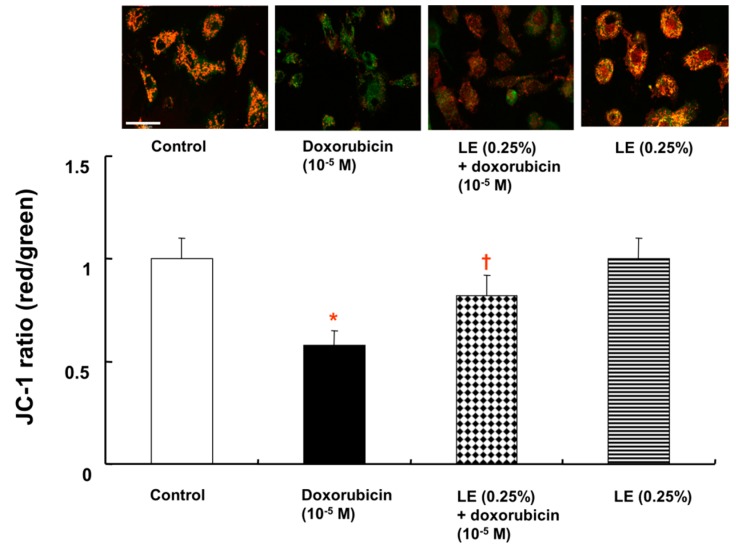
Effects of Intralipid^®^ (lipid emulsion, LE) on the doxorubicin-induced decrease in the mitochondrial membrane potential (MMP) in H9c2 rat cardiomyoblasts. JC-1 is a monomer that exhibits green fluorescence at low MMP and red fluorescence at high MMP. Mitochondria with an intact MMP show red fluorescence. Scale bar: 50 µm. Data (N = 3) are presented as the mean ± SD. * *p* < 0.001 versus control. † *p* < 0.001 versus doxorubicin alone.

The MMP was analyzed by determining the ratio of JC-1 dye fluorescence. The doxorubicin only-treated cells exhibited a higher percentage of green fluorescence (JC-1 ratio: 0.58 ± 0.07) than cells pretreated with lipid emulsion followed by doxorubicin (JC-1 ratio: 0.82 ± 0.10), which exhibited a higher percentage of red fluorescence than green fluorescence (Figure 6). However, the JC-1 ratio in cells treated with lipid emulsion alone (JC-1 ratio:1 ± 0.10) was similar to that in control cells (JC-1 ratio:1 ± 0.10), and cells treated with lipid emulsion alone exhibited a higher percentage of red fluorescence than green fluorescence (Figure 6). The JC-1 monomer emits green fluorescence, which indicates a decrease in MMP and mitochondrial dysfunction. These results confirmed that a doxorubicin-induced decrease in MMP can be prevented by lipid emulsion pretreatment by decreasing the number of dysfunctional mitochondria in cardiomyocytes.

## 4. Discussion

**Figure 7:**
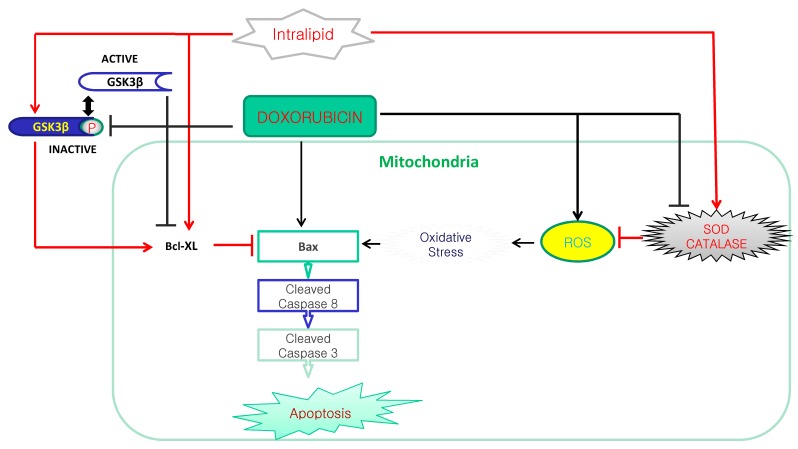
The putative cellular mechanism associated with the lipid emulsion (Intralipid^®^)-mediated attenuation of apoptosis induced by a toxic dose of doxorubicin in H9c2 rat cardiomyoblasts. ROS: reactive oxygen species. SOD: Superoxide dismutase. GSK-3β P: Phosphorylated glycogen synthase kinase-3β.

This is the first study to suggest that lipid emulsion attenuates the doxorubicin-induced late apoptosis of rat cardiomyoblasts, and this effect appears to be associated with the inhibition of oxidative stress (Figure 7). The major findings of this in vitro study are as follows: (1) Lipid emulsion inhibited the cell viability reduction induced by doxorubicin; (2) lipid emulsion inhibited the late apoptosis induced by doxorubicin; and (3) lipid emulsion inhibited the increased ROS and MDA induced by doxorubicin, whereas lipid emulsion pretreatment reversed the decreased SOD and catalase activity and the reduced MMP induced by doxorubicin.

Extensive efforts have been dedicated to reducing doxorubicin-induced cardiotoxicity to improve the therapeutic potential of doxorubicin; however, due to the complex molecular mechanism underlying doxorubicin-induced cardiotoxicity, limited success in reducing its toxicity to cardiomyocytes has been achieved [18,19,20,21]. The two main mechanisms considered to be important in doxorubicin-induced cardiotoxicity are oxidative stress and cardiomyocyte apoptosis [2,22,23], the latter of which contributes to heart failure [24,25]. The mechanism associated with the apoptosis observed in doxorubicin-induced cardiotoxicity has been extensively investigated, and multiple pathways are known to be involved in apoptotic cell death [16,26]. Lipid emulsion reversed the cell viability decrease induced by bupivacaine or verapamil in H9c2 cells [13,14]. Similar to these previous reports, the results presented herein demonstrated that lipid emulsion attenuated the decreased cell viability induced by doxorubicin, as determined by the MTT assay (Figure 1B) [13,14]. α- and γ-linolenic acid, which are long-chain fatty acids, counteract the cardiotoxicity of doxorubicin [10,27]. However, this is the first study to employ lipid emulsion as a whole to counterbalance doxorubicin-induced cardiotoxicity in H9c2 cells. The long-chain fatty acids present in Intralipid^®^ (with 100% long-chain fatty acids) include 53% linoleic acid, 24% oleic acid, 11% palmitic acid, 8% α-linolenic acid and 4% stearic acid, but which long-chain fatty acid is mainly involved in the lipid emulsion-mediated attenuation of doxorubicin-induced decreased cell viability in H9c2 rat cardiomyoblasts remains to be determined [7].

Lipid emulsion attenuates the increased expression of caspase-8 and/or Bax induced by verapamil, bupivacaine and malathion in rat cardiomyoblasts and lung tissue [12,13,14]. In addition, lipid emulsion attenuates apoptosis induced by toxic doses of bupivacaine and verapamil in H9c2 rat cardiomyoblasts [13,14,28,29]. Similar to previous reports, Annexin-V-FITC/PI staining revealed that doxorubicin alone induced late apoptosis in H9c2 cells, and pretreatment with lipid emulsion decreased the late apoptosis induced by doxorubicin, which was further confirmed by the TUNEL assay [13,14,28,29]. Thus, our current results demonstrate that lipid emulsion have a protective effect on the late apoptosis induced by doxorubicin in H9c2 rat cardiomyoblasts. An in-depth evaluation of the apoptotic signaling pathway revealed that doxorubicin initiates apoptosis by increasing the expression of the intrinsic proapoptotic protein Bax and the extrinsic proapoptotic protein cleaved caspase-8, which stimulates the caspase cascade, ultimately activating cleaved caspase-3 and inducing cell death [30]. In contrast to the effects of doxorubicin alone, lipid emulsion pretreatment activates the anti-apoptotic protein Bcl-XL and inhibits the expression of the proapoptotic protein Bax, thereby suppressing apoptosis and rescuing the cell from programmed cell death, as evidenced in our present findings. GSK-3β activates the proapoptotic protein Bax and capsase-3 in mitochondria, which leads to apoptosis [31]. However, GSK-3β phosphorylation attenuates mitochondrial apoptosis via attenuation of cytochrome c release [31]. Pretreatment with the GSK-3β inhibitor SB216763 followed by doxorubicin partially reversed the decreased cell viability induced by doxorubicin alone (Figure 1D). Furthermore, the combined treatment with lipid emulsion and doxorubicin had no significantly different effects on the cell viability, as compared with the combined treatment with SB216763, lipid emulsion and doxorubicin (Figure 1D). These results suggest that doxorubicin-induced decreased cell viability is partially mediated by GSK-3β activation. Consistent with the cell viability results of the current study, lipid emulsion reversed the decreased GSK-3β phosphorylation induced by doxorubicin (Figure 3E), which is associated with GSK-3β inactivation and may subsequently lead to anti-apoptosis. Considering a previous report, the lipid emulsion-mediated reversal of the decreased GSK-3β phosphorylation induced by doxorubicin may partially contribute to lipid emulsion-mediated anti-apoptosis via decreasing the Bax/Bcl-XL ratio [31].

Doxorubicin is known to cause oxidative stress-mediated cardiotoxicity via the formation of ROS and induction of cardiac dysfunction [32]. Doxorubicin is one hundred times more concentrated in mitochondria than in plasma. Redox cycling of doxorubicin in mitochondria generates high levels of ROS, including superoxide anions and hydrogen peroxide, causing oxidative stress in cardiomyocytes and leading to cell damage [6,32]. Oxidative stress is associated with the imbalance induced by excessive oxygen free radicals that exceed the antioxidant response [33]. Cell membranes are composed of polar lipids and their peroxidation, which is due to the attack of excessive ROS on polyunsaturated fatty acids, results in cell damage and death [34]. Oxidative stress in the cell causes high lipid peroxidation, resulting in the release of MDA, the product of lipid peroxidation by free radicles [34]. Excessive ROS production induces apoptosis via interacting with critical signaling molecules, including apoptosis signal-regulated kinase [33]. Consistent with a previous report, doxorubicin increased ROS production, as shown in Figure 4A,B, whereas lipid emulsion attenuated the increased production of ROS induced by doxorubicin (Figure 4A,B) [35]. MDA was increased by doxorubicin (Figure 5A), whereas lipid emulsion pretreatment inhibited the increased MDA induced by doxorubicin (Figure 5A). Lipid emulsion attenuates the increased MDA concentration and ROS production induced by bupivacaine, which leads to attenuation of apoptosis [29]. Similar to this previous report, the results presented herein suggest that lipid emulsion attenuates the oxidative stress induced by doxorubicin, which appears to contribute to the inhibition of apoptosis induced by doxorubicin in H9c2 rat cardiomyoblasts [29]. In addition, similar to this result, α-linolenic acid—a long-chain fatty acid found in Intralipid^®^—inhibits cardiotoxicity by decreasing oxidative stress and apoptosis [10]. As α-linolenic acid partially inhibited the increased amount of ROS evoked by doxorubicin (Figure 4C), the lipid emulsion-mediated inhibition of ROS increased by doxorubicin may be partially due to the α-linolenic acid contained in Intralipid^®^ [7]. ROS induced by doxorubicin-induced oxidative stress in cardiomyocytes are controlled by antioxidant enzymes, including SOD, catalase, and glutathione peroxidase [36,37]. SOD and catalase inhibit apoptosis by removing superoxide anions and hydrogen peroxide, respectively [38]. Lipid emulsion was reported to reverse the inhibited activity of SOD or catalase induced by bupivacaine and malathion, leading to anti-apoptosis and decreased lung injury [12,29]. Consistent with these previous reports, the lipid emulsion-mediated reversal of the decreased activity of SOD and catalase induced by a toxic dose of doxorubicin observed in the current study appears to contribute to the anti-apoptosis of rat cardiomyoblasts [12,29].

Intrinsic mitochondrial apoptosis leads to mitochondrial membrane depolarization and mitochondrial outer membrane permeabilization, and doxorubicin reduces the MMP and calcium retention capacity [39,40]. Combined treatment with lipid emulsion and bupivacaine increases the MMP more than bupivacaine alone [29]. In addition, the mitochondrial respiratory function in the cardiac depression induced by bupivacaine is enhanced by lipid emulsion [41]. Consistent with these previous reports, doxorubicin decreased the MMP in H9c2 rat cardiomyoblasts, whereas lipid emulsion reversed the attenuated MMP induced by doxorubicin (Figure 6) [29,40].

As lipid emulsion has been clinically used for parenteral nutrition, lipid-soluble drug delivery, and the treatment of drug toxicity without inducing severe side effects, lipid emulsion may be able to be clinically used to attenuate the cardiotoxicity induced by doxorubicin [7,11]. However, extrapolation of the results of this in vitro study to clinical applications has some limitations. First, the median plasma concentration of doxorubicin and the steady-state plasma concentration of doxorubicin in patients treated with doxorubicin are approximately 10^−7^ and 4.3 × 10^−7^ M doxorubicin, respectively [32,42]. The supraclinical concentration (10^−5^ M) of doxorubicin used in the current study, which induced apoptosis in rat cardiomyoblasts, may be encountered in cardiac mitochondria because doxorubicin-induced cardiac toxicity is associated with a cumulative dose of doxorubicin, and the doxorubicin concentration in cardiac mitochondria is one hundred times more than that in extracellular fluid [6,43]. However, lipid emulsion may also inhibit the therapeutic effect (for example, apoptosis) of doxorubicin on cancer cells. As the effect of lipid emulsion on doxorubicin-induced apoptosis may be dependent on the type of cell, organ and concentration of doxorubicin, further study to examine whether lipid emulsion interferes with the antitumor activity of doxorubicin in cancer cells is needed. Second, pretreatment with lipid emulsion attenuated the doxorubicin-induced apoptosis of cardiomyoblasts in the current study, whereas posttreatment with lipid emulsion is clinically used to alleviate cardiotoxicity followed by toxic doses of local anesthetic or other drugs. Third, this study used H9c2 rat cardiomyoblasts instead of human cardiac myocytes.

## 5. Conclusions

In conclusion, these results suggest that lipid emulsion inhibits the late apoptosis induced by a toxic dose of doxorubicin in H9c2 rat cardiomyoblasts. Lipid emulsion attenuates the formation of ROS and increases the antioxidant enzyme activity in mitochondria. Anti-apoptotic protein Bcl-XL expression was vital for cardiomyoblast survival during doxorubicin treatment, and Bcl-XL protein expression was enhanced by lipid emulsion treatment, thereby reducing the toxic effect of doxorubicin in rat cardiomyoblasts.
